# HPV knowledge, screening barriers and facilitators, and sources of health information among women living with HIV: perspectives from the DC community during the COVID-19 pandemic

**DOI:** 10.1186/s12905-022-01689-y

**Published:** 2022-04-09

**Authors:** Annie Coriolan Ciceron, Min Jeong Jeon, Anne Kress Monroe, Michelle Elise Clausen, Manya Magnus, Daisy Le

**Affiliations:** 1 Department of Policy, Populations, and Systems, School of Nursing, The George Washington University, 1919 Pennsylvania Avenue NW, Suite 500, Washington, DC 20006 USA; 2 Department of Epidemiology, Milken Institute School of Public Health, The George Washington University, 950 New Hampshire Avenue NW, 5th Floor, Washington, DC 20052 USA; 3 Department of Prevention and Community Health, Milken Institute School of Public Health, The George Washington University, 950 New Hampshire Avenue NW, 3rd Floor, Washington, DC 20052 USA; 4 George Washington University Cancer Center (GW Cancer Center), The George Washington University, 800 22nd Street NW, Suite 7000C (Science and Engineering Hall), Washington, DC 20052 USA

**Keywords:** HPV knowledge, Screening, Sources of health information

## Abstract

**Background:**

High-risk human papillomavirus (HPV) causes 99% of cervical cancer cases. Despite available prevention methods through the HPV vaccine and two screening modalities, women continue to die from cervical cancer worldwide. Cervical cancer is preventable, yet affects a great number of women living with HIV (WLH). Low screening rates among WLH further exacerbate their already high risk of developing cervical cancer due to immunosuppression. This study explores WLH’s current cervical cancer knowledge, screening barriers and facilitators, and sources of health information.

**Methods:**

Focus group discussions were conducted with 39 WLH aged 21 years old or older, who resided in the Washington-Baltimore Metropolitan Area. Emergent themes were classified and organized into overarching domains and assembled with representative quotations.

**Results:**

The women had limited knowledge of HPV and the cervical cancer screening guidelines for WLH. Coronavirus 2019 (COVID-19) pandemic has amplified screening barriers due to decreased accessibility to usual medical appointment and cervical cancer screenings. Screening facilitators included knowing someone diagnosed with cervical cancer and provider recommendations. WLH indicated that they obtained health information through in-person education (providers, peer groups) and written literature. Due to the pandemic, they also had to increasingly rely on remote and technology-based communication channels such as the internet, social media, television, radio, email, and short message service (SMS) text messaging.

**Conclusions:**

Future health interventions need to explore the possibility of sharing messages and increasing cervical cancer and HPV knowledge of WLH through the use of SMS and other technology-based channels.

## Background

In the United States (U.S.), it is estimated that about 14,100 women will be diagnosed with invasive cervical cancer (ICC) in 2022 [1]. Persistent infection with high-risk human papillomavirus (HPV) causes 99% of cervical cancer cases [2]. Despite available prevention methods through the HPV vaccine and two screening modalities (i.e., the Papanicolaou (Pap) test/smear and the HPV test), approximately 4280 women are projected to die in the United States in 2022 [1]. In particular, women living with HIV (human immunodeficiency virus) (WLH) are disproportionately affected by ICC. Compared to women without HIV, WLH have a fourfold excess risk of developing dysplasia largely due to immunosuppression caused by their HIV status [3] and are more likely to have persistent HPV infection with increased progression to high-grade cervical intraepithelial neoplasia and ICC [2, 4–6].

Cervical cancer is preventable, and yet affects a great number of WLH due to low screening rate. Current guidelines recommend that Pap screening for WLH should begin no later than age 21 or within one year of the onset of sexual activity. For those who are not immunocompromised, pap screening begins at 25 years of age [7]. A recent study on a longitudinal cohort of individuals living with HIV from the District of Columbia (DC; the District) found that among WLH who were screening-eligible and had no history of cervical cancer, only 43% were screened for cervical cancer per the HIV Medicine Association (HIVMA) of the Infectious Diseases Society of America (IDSA) screening guidelines [5]. It has also been similarly reported in a 10-year longitudinal study (2005–2014; Baltimore, MD) that some WLH were also not receiving Pap tests at the recommended frequency with longer periods in between Pap test intervals [8]. While 79% of WLH received Pap testing, 21% did not receive follow-up Pap testing, and only 11% received testing as recommended [8].

Knowledge of screening can positively affect screening intention and behavior [9]; yet few U.S.-based studies have explored WLH’s HPV and cervical cancer knowledge [10]. Of the four U.S.-based knowledge-focused studies (five articles) that were included in Wong and colleague’s recent scoping review, two (South Florida and Alabama-based) used qualitative methods of focus groups or individual interviews to explore knowledge and perceptions of HPV and cervical cancer screening [11, 12], and two (Florida and Southeastern-based) used questionnaires to examine knowledge, attitudes, perceptions, and screening behaviors [13–16]. These studies highlight the significance of and need for increasing knowledge and awareness related to HPV [11, 13, 17], cervical cancer screening recommendations [12, 18], and cervical cancer prevention [12, 19] among WLH. However, the generalizability of findings from these studies are limited in several respects. First, all four of the U.S.-based studies were conducted among WLH population in the South region of the U.S. [11, 12, 14–16]. In addition, the populations of interest in both of the qualitative studies were restricted to specific racial/ethnic groups and reflected WLH who were already receiving care [11, 12].

Recognizing that there are geographical variation in the prevalence of HIV and cancer, we sought to explore the knowledge of cervical cancer prevention methods and screening barriers and facilitators among WLH from the Washington-Baltimore Metropolitan Area (WBMA); a topic that should be explored further since residents of DC are more at risk for HIV infection and HIV diagnosis in their lifetime compared to the average American [20, 21], and cervical cancer has recently been reported as the second most common incident AIDS-defining cancer (0.7 per 1000) among a cohort of WLH from DC [5].

The current study, therefore, aimed to provide a different perspective in that it reflects on the views of WLH who are specifically from a mid-Atlantic metropolitan region where there is a high HIV prevalence rate. The study was conducted during the early phases of the Coronavirus 2019 (COVID-19) pandemic in the U.S.; during which remote and telehealth medical services had to be quickly adapted by healthcare providers [22]. Therefore, study results provide critical insight into how knowledge influences screening behavior (including access); how best to develop and support health programs and interventions that aim to increase cervical cancer knowledge and screening uptake among a unique population group at risk; but also recommendations that take into account the new realities of healthcare delivery during the COVID-19 pandemic, an unprecedented time in our history.

## Methods

### Recruitment, sample, and ethical approval

This qualitative study was part of a larger mixed-method study that explored the acceptability of self-collecting cervicovaginal samples among WLH. Six focus group discussions (FGD) with 39 eligible WLH (mean: 6.5 individuals/FGD; range 5–9 individuals/FGD) were organized to discuss HPV knowledge, cervical cancer screening barriers and facilitators, and sources of health information among other WLH in the WBMA. None of the women participated in more than one FGD. Purposive sampling was used to engage community stakeholders serving WLH and convenience and snowball sampling methods were used to recruit WLH. Organizations that support WLH were contacted to post and distribute flyers to potential participants. ResearchMatch, a national health volunteer registry supported by the U.S. National Institutes of Health as part of the Clinical Translational Science Award program, was also used to recruit WLH.

Eligible participants for the FGD included WLH who were 21 years old or older, resided in the WBMA (Washington DC, Maryland, Virginia), and were not currently participating in any other studies focused on HPV self-sampling. Participants’ eligibility was not restricted to any specific racial/ethnic group and a community-based open recruitment approach was adopted to intentionally allow for enrollment of WLH who were already receiving routine care, as well as those who were not. Participants’ eligibility was ascertained over the phone and online using the Research Electronic Data Capture (REDCap) web application [23]. Eligible participants were then mailed and asked to return their signed informed consent to the research team in a pre-paid envelope before their scheduled FGD. This research was reviewed and approved according to the George Washington Institutional Review Board’s procedures for research involving human subjects (NCR191689).

### Data collection

Virtual FGD took place between April and June 2020. FGD topics included: (1) cervical cancer prevention knowledge and screening behavior; (2) facilitators and barriers to cervical cancer screening uptake (e.g., insurance and income limitations, relationship with doctors and other healthcare providers, limited access due to the COVID-19 pandemic); and (3) sources of health information. Upon FGD completion, all participants were mailed their $25 gift card incentive, as well as a packet with information on HPV and cervical cancer prevention and a local resource directory listing options for screening with or without insurance. Due to the COVID-19 pandemic, and inability to meet with participants in-person, the one-hour discussions were pre-scheduled and conducted remotely via WebEx in audio only, to protect confidentiality. Trained moderators and research staff facilitated the discussions. Upon entry into the WebEx room, participants were each assigned numbers that served as personalized identifiers throughout the discussion. They were asked to announce their assigned number before speaking, thus their personal identity would not be captured in the audio recordings. All discussions were digitally recorded with the participants’ approval.

### Data analysis

All discussion sessions were transcribed, coded, and analyzed using thematic analysis [24]. Two experienced researchers (DL, AC) served as the lead for the data analysis. After each transcript was individually reviewed for accuracy, the researchers met to discuss and develop a primary list of codes. Codes were defined, symbolic passages were identified, and the inclusion and exclusion criteria were designed. Two additional coders (MJJ, LG) were trained in the codebook and codes were applied independently once inter-rater reliability was reached. NVivo software (Version 12 Plus) was used to code themes and validate the application of the codes. Emergent themes were classified and shared between reviewers (DL, AC, MJJ, LG) and themes were organized into overarching domains and assembled with representative quotations. Discrepancies were resolved through discussion in a process of constant comparison until inter-rater reliability was reached (Kappa coefficient > 0.80). Descriptive analyses were conducted using SPSS 26.0 to describe the study sample, including information related to their DC residence, most specifically distribution by Wards to ensure representativeness. In the District, there are eight jurisdictions known as Wards (numbered 1–8)—each represented by its own council member. Wards 4, 5, 7, and 8, have the highest rates of HIV and the highest concentrations of residents of color [20, 21].

## Results

### Sociodemographic characteristics

As presented in Table 1, FGD participants included WLH between the ages of 35 and 66 years old. Half (50.0%) of the women interviewed had some college or technical school, and a half (50.0%) were receiving disability benefits. The majority (87.2%) of the women were residents of the District, representing Wards 8 (40.6%), 7 (15.6%), 1 (12.5%), and 5 (also 12.5%). Wards 7 and 8 have the lowest socio-economic status, and the highest unemployment level [25].

**Table 1:** Characteristics of women living with HIV (N = 39)

Characteristics of FGD participants	Number (percent)
*Age, years*	
21–30	0 (0%)
31–40	3 (7.7%)
41–50	7 (17.9%)
51–60	20 (51.3%)
61–70	9 (23.1%)
Median (range)	55 (35–66)
*Highest education (n* = *35)*	
Elementary	1 (2.8%)
Some high school	5 (13.9%)
High school graduate	11 (30.6%)
Some college or technical school	18 (50.0%)
Employment*	
Disabled (n = 36)	18 (50.0%)
Not currently working for pay (n = 36)	11 (30.6%)
Part-time (n = 36)	5 (13.9%)
Full-time (n = 36)	3 (8.3%)
Residing in DC**	34 (87.2%)
Ward 1	4 (12.5%)
Ward 2	2 (6.3%)
Ward 4	1 (3.1%)
Ward 5	3 (9.4%)
Ward 6	4 (12.5%)
Ward 7	5 (15.6%)
Ward 8	13 (40.6%)
Health insurance (n = 36)	34 (94.4%)
Had a usual source of care (n = 36)	33 (91.7%)
Had an history of cervical cancer or hysterectomy	10 (25.6%)
Ever had a Pap test (n = 36)	34 (94.4%)
Had a Pap test in the past 12 months (n = 36)	28 (77.7%)

^*^Participants could select more than one option

^**^For municipal purposes, including local elections and city planning, Washington, D.C. is divided into eight wards—each represented by its own councilmember

Almost all of the WLH indicated that they were currently insured (94.4%) and had a usual source of care (91.7%). A quarter (25.6%) of the women indicated that they had a history of cervical cancer or hysterectomy. As it relates to their cervical cancer screening history, 94.4% indicated that they have ever had a Pap test in their lifetime, and 77.7% indicated that they had a Pap test in the last 12 months.

### Overview of key themes

**Fig. 1:**
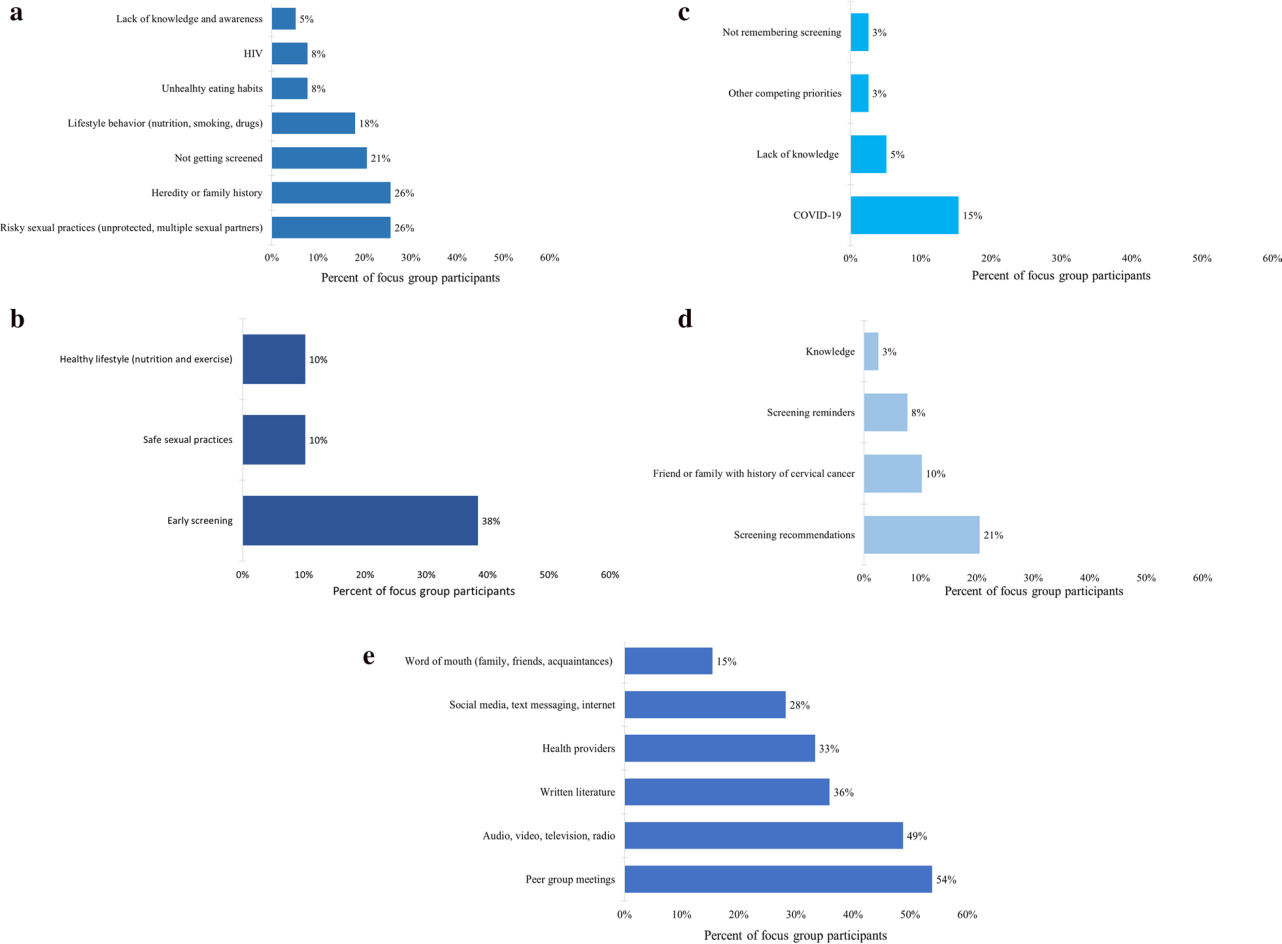
Frequency of themes shared by FGD participants on cervical cancer and HPV prevention knowledge, screening barriers and facilitators, and sources of health information. **a** Cervical cancer and HPV risk factors. **b** Cervical cancer and HPV prevention. **c** Barriers to cervical cancer screening. **d** Facilitators to cervical cancer screening. **e** Usual sources of health information (prior to the COVID-19 pandemic)

A visual/graphical representation of the key themes, raised during the FGD sessions by WLH, is presented in Fig. 1.

Based on the six FGD, four thematic categories emerged (Table 2).

**Table 2:** Key themes and representative quotes from focus group discussions

Theme	Representative quote
*Cervical cancer and HPV knowledge*	
Risk factors	“With HIV, I believe that we are more prone to infections, so there is more change of getting the cervical cancer” “I also think those have multiple sexual partners without using proper protection …”
Prevention	“I think we should get vaccinated for HPV, we should practice safe sex.” “Eat heathy. If you’re having sexual activities, protect yourself and proper rest […], exercise, just take care of your body.” “Regular checkups for Pap smears […] every 6 months.”
*Barriers and facilitators to cervical cancer screening*	
Facilitators and barriers to screening	“They are used to taking care of other people and neglect themselves, its’ not a question that they don’t want to take it, they just don’t think about taking it.” “There’s very little information that’s put out there for us to learn about it.” “They will call me and say it's time.”
Screening recommendations	“Um, the main reason is because there was, well it was requested from my doctor.”
Screening behaviors	“I do it annually but if something is going on, I’m donna call the doctor and go see what’s going on” “[I get screened] when something’s wrong […].”
COVID-19	“Right now, it’s just in the middle of my list because I can’t go to the doctor right now.”
*Avenues to increase knowledge and adherence to cervical cancer screening*	
Written literature	“Most of my info came from HPV info pamphlets […] that’s how I learned about cervical cancer.” “We have our groups and stuff. We take notes down and they give us flyers, you know, different information, you know, on different things, you know, to keep us knowledge too and then I always keep my flyers and stuff and read over them. You know, different little things. I want to know and maybe some of it to share with other people.”
Peer group meetings	Um, you know how in our HIV community, we have pharmaceutical reps that come out and do presentations? That needs to be implemented on a regular basis, pharmaceutical company to come out and have a women's meeting, have a nice meal, a nice dinner, uh, semi-dress up and get some education and feel good about yourself while you're getting the education.” “I get a lot of my information through focus groups and studies”
Text messaging	I didn't, I don't know a lot of things about it, it's what I've read in the pamphlet and I've read and talked to my doctor about, you know. But, texting would be, you know, a good idea to do.” “Yeah, that would be good also, ‘cause a lot of stuff now days is given, is sent through texts also so that would be interesting.”
*Impact of the COVID-19 pandemic on sources of health information*	
Group sessions	“We used to have groups in, um, have people consistently coming into class and teaching us […] I haven't been in my group in probably like a good 3 or 4 months.” “Well actually the same as the other, um, participants, at focus groups, support groups, and then they've got a lot of meetings on Zoom, like we're doing now. I think that's the best way right now because of what's going on.”

### Cervical cancer and HPV knowledge

Specifically in terms of their general knowledge of cervical cancer and HPV, most of the women accurately identified the risk factors for cervical cancer citing lifestyle behavior such as smoking and having unprotected sex (see Fig. 1a). They also recognized that having HIV and not getting Pap tested as recommended increases their risk for cervical cancer (“With HIV, I believe that we are more prone to infections so there is more chance of getting the cervical cancer”). Prevention methods that were identified by the women included safe sexual practice, healthy eating, exercising, and getting the HPV vaccine (see Fig. 1b). A few of the women shared that they did not think cervical cancer was preventable.

While the participants conveyed an adequate knowledge of cervical cancer, many did not know about HPV. Some of the women mentioned that they either had never heard of HPV or had heard of it but had no additional knowledge beyond that. A few of the women expressed that they were only familiar with the term “HPV” because they had recently been exposed to the HPV vaccine advertisements through billboards, radio, and television but that they did not realize that the HPV was sexually transmittable (“I don’t remember being told that it was transmitted from sex, this is the first [time] I’ve heard it”). Cervical cancer screening knowledge was also low among our participants. The women could not explain what the Pap test is and what it entails; e.g., some women incorrectly associated the Pap smear with the general testing for STDs (as opposed to the identification of cell changes or abnormal cells in the cervix). It was also unclear to our participants as to when a first Pap smear should be initiated: some mentioned that it should be initiated upon a woman’s first menstrual cycle. Finally, although our participants knew that cervical cancer screening guidelines differed for WLH, many were unsure of the specific guidelines.

### Barriers and facilitators to cervical cancer screening

When prompted about barriers to cervical cancer screening (see Fig. 1c), women in our study expressed being less likely to get screened due to their lack of knowledge about cervical cancer (“There’s very little information that puts out there for us to learn about it”), other competing priorities (such as having to take care of their family), not remembering about the screening, and the inability to go to their usual check-ups due to the COVID-19 pandemic. Because of the COVID-19 pandemic, they voiced that they did not feel safe going into their provider’s office unless it was for an emergency (“Um, going into the office right now for Pap smear and stuff like that is very dangerous, so we really need one you can do at home.”)*.* Some even expressed that if it was not offered by their provider, they would not request the screening unless they were experiencing abnormal symptoms.

Screening facilitators identified (see Fig. 1d) were being more educated about cervical cancer risk factors, and their susceptibility to HPV as a WLH: “It is easier to get some infection even if I’m taking my medicine as usual, so it’s a priority to get a Pap smear test every time required.” They also indicated that having a family history of cervical cancer, or knowing someone affected by cervical cancer made them more aware of cervical cancer and more likely to adhere to the recommended screenings (“My sister died from it. Um, I get checked up for it, but um, I had high grade lesions and, um, I have my cervix removed.”)*.* Among the women who indicated that they received a Pap test in the previous 12 months, many directly attributed their screening adherence to direct recommendations from their provider (“I need to do that, um, but you know, it’s kind of hard with coronavirus right now. So, um, but usually I’m motivated by my doctor, the Gyno”)*.* They mentioned receiving reminder notices (mail or calls) from their providers when they are due for their next screening (“I get a letter in the mail a week before it’s supposed to be done and then I get a notice, […] they give me an extra call cause they know I don’t like them”).

### Avenues to increase knowledge and adherence to cervical cancer screening

To increase understanding of how knowledge gaps can be addressed among the target population, we asked women to share their usual source of health information, and health education preferences (see Fig. 1e). The women listed that they obtained their health information (general, cervical cancer, and HPV-focused) through various channels: in-person education with their providers, conversations with peers or in group settings such as support groups, focus group discussions for research studies, community/organization-initiated workshops (“A lot, I get a lot of my information through focus groups and studies and everything.”). Some women listed that they also obtained their health information through written literature such as pamphlets, though they also acknowledged that literacy level needs to be considered (“I think they should break it down a little more clear when they do the pamphlet for cancer”), and that some may prefer pictorial messages (“So, I think a picture’s always good for the person who can’t read as good as someone else or has problems”).

### Impact of the COVID-19 pandemic on sources of health information

Due to the COVID-19 pandemic, the women noted that they could no longer have those in-person education sessions. As most of the sessions have ceased or migrated to an online platform, they had to quickly transition to and rely on remote and technology-based communication channels (“We are unsure about how long this COVID-19 thing is going on, so I’m comfortable with doing video calls and phone calls from my doctor instead of actually going into the office.”). Other forms of media channels raised by the women were the internet, television, radio, email, text messaging (“You know, the focus group and […] support groups over Zoom, and that’s a good way to get out the information, of course emails, texts because there’s a lot of women that don’t know about this.”), videos, advertisement, and social media (“Yes, social media, word of mouth, because you know, […] we’ve been together of years so, we network together so we know different things, we communicate with each other and we pass on messages and things like that. When one person tells one person, we find out together and do things together to find out things like that.”)*.* Although some women recognized that messages that used fear tactics could work for some, they stressed that messages conveying a sense of urgency were also effective (“Not really fear, but concern, a message of concern and how, how needed it is for you to know about HPV.”).

## Discussion

This study adds further evidence to the body of literature concerning knowledge of HPV and cervical cancer, facilitators and barriers to cervical cancer screening (as amplified by the COVID-19 pandemic), as well as current sources of and preferred avenues for health information among WLH. Participants had limited knowledge of HPV and the cervical cancer screening guidelines for WLH. It was also unclear to our participants as to when a first Pap smear should be initiated: some mentioned that it should be initiated upon a woman’s first menstrual cycle, whereas the Guidelines for the Prevention and Treatment of Opportunistic Infections in Adults and Adolescents with HIV [26] currently recommends that Pap screening for WLH should commence within one year of the onset of sexual activity regardless of the mode of HIV transmission (e.g., sexual activity, perinatal exposure) but no later than age 21. Screening barriers included limited access to medical appointment due to COVID-19 pandemic, a barrier that could be attenuated with remote-based HPV self-sampling interventions where WLH could collect their sample in the comfort of their home and return it to the provider or laboratory [27]. The WLH also indicated that they were more likely to get screened for cervical cancer if recommended by their provider. The women indicated that while they had previously obtained their health information through in-person education (providers, peer groups) and written literature, they have had to increasingly rely on remote and technology-based communication channels such as the internet, social media, television, radio, email, SMS text messaging, due to the COVID-19 pandemic.

The majority of WLH in this study indicated that they had been screened in the past 12 months, whereas in the Alabama Cervical Cancer Health Project [12] only half of the participants were screened. When discussing what prompted the participants in the current study to get screened, the women indicated that having a friend/family who had cervical cancer or an abnormal Pap test played an important role in motivating them to get screened regularly. This finding on adherence to screening among WLH from the WBMA aligns with Wigfall and colleagues’ conclusions that those who had someone within their social networks with a history of an abnormal Pap test were indeed more knowledgeable about cervical cancer, which may explain their adherence to screening [15]. WLH with friends/family who had an abnormal Pap test were also found to be more informed of the cervical cancer screening guidelines for WLH than women who did not [15]. However, their knowledge on the recommended follow-up care after abnormal Pap test results varied [11, 12, 15].

As many of the women have conveyed, knowledge of HPV and cervical cancer and their preventative measures are essential to increase health-seeking behaviors, such as routine screening. Earlier studies suggest that, worldwide, WLH have limited knowledge on what HPV is, the method of transmission [7, 10, 11, 13, 17], cervical cancer screening recommendations for WLH [10, 12], prevention methods [10, 12, 19], and the association between HIV, HPV, and cervical cancer [10, 13]. The limited knowledge regarding cervical cancer and HPV among WLH in the current study provides additional insight into why most of them indicated that they would not seek out cervical cancer screenings unless offered by their health care providers. The majority of the women in this study had access to care (i.e., a usual source of care, health insurance) and reported having a Pap test done within the last year. Though they had been screened, they attributed this mainly to their provider’s recommendation. These findings are congruent with other studies that have found that WLH complied with their providers’ screening recommendations even when they did not understand the procedures [16]. The women’s lack of understanding of the tests (i.e., purpose, methodology, and screening guidelines) [11, 16], however, has been cited as a barrier that would most likely impede them from maintaining the health behavior over time [10].

When exploring other avenues where the women in this study received their health information, they placed great emphasis on group meetings and forums where they had expert speakers present on a specific topic, allowing them to learn and discuss new information related to their health. This finding is in line with the South Florida study among Haitian immigrants where the women also expressed their preference for group and one-on-one educational sessions [11]. While findings from the same study suggested that text-based educational materials were not favorable [11] among their study sample due to limited literacy and health literacy, participants in this current study conversely expressed print materials (among other media and technology-based sources such as television and radio) as their current sources of and preferred avenues for health information. They further expressed that SMS text messaging would be a viable and important communication channel to adopt in order to widely reach and increase cervical cancer and HPV knowledge among WLH in the WBMA. These remote communication strategies have the potential to increase cervical and HPV knowledge, and help improve cervical cancer screening rates among WLH in the WBMA.

### Limitations

Findings from the current study may not be necessarily generalizable to WLH who do not consistently interact with the health care system or are uninsured. The District of Columbia has the second lowest uninsured rate in the nation, with nearly 97% of residents being covered [28]. Since the majority of the participants had health insurance or usual source of care, they may have been more likely to adhere to their cervical cancer screenings due to their providers’ prompting or recommendation. Additionally, this study reflects the views of WLH from an urban mid-Atlantic metropolitan geographical region and may not be generalized to WLH from rural areas and/or have lower educational attainment (over 80% of our participants had a high school diploma or higher). Because the majority of the participants were older than 50 years of age, findings on screening facilitators and barriers and sources of health information may also differ for a younger group of WLH.

We conducted our FGD using the WebEx’s voice-only feature; therefore, the facilitator could not rely on visual cues that could have added more context to the discussions. This method may have also decreased the level of rapport that face-to-face interactions would have helped the facilitator build with the participants. Nevertheless, the discussion itself was not the first time through which the participants had gotten an opportunity to interact with the study team; each participant had previously spoken with the facilitator or another team member at least once on the phone (e.g., through the study’s initial recruitment and eligibility screening processes or through a follow-up phone call reminding them about their upcoming FGD session). Through early consultation with key community stakeholders, our team purposefully selected the voice-only method to account for the digital divide that may exist among our participants [29], including connectivity issues, and no access to a computer or other digital device with a camera (tablet computer, smartphone, etc.).

### Implications for policy and/or practice

This study reaffirmed the importance of knowledge of prevention methods in improving cervical cancer screening among WLH. To this end, additional educational and communication channels need to be considered for WLH who are not in care, do not attend group meetings, and do not have a community within the health care system. Other channels need to also be considered to account for situations similar to what we are currently going through with COVID-19, where in-person group meetings and interactions may remain limited over a longer period of time. Future studies should explore the feasibility and efficacy of SMS text messaging to widely reach and to increase HPV and cervical cancer prevention knowledge and awareness among harder-to-reach at-risk communities such as WLH.

## Conclusions

Results from this study highlighted knowledge gaps, screening barriers, and facilitators to cervical cancer screening which can be addressed through comprehensive health interventions. These interventions should take into account WLH’s current knowledge of cervical cancer prevention and their usual sources of health information to improve their knowledge of HPV and cervical cancer prevention methods. Improved cervical cancer screening knowledge can, in turn, lead to increases in screening intention and behavior [9]. Future health interventions need to explore the possibility of sharing messages and increasing cervical cancer and HPV knowledge of WLH through the use of SMS text messaging and other technology-based channels. These tools will be valuable in situations where in-person interactions are limited, as seen during the COVID-pandemic. Access to screening can also be improved through remote-based interventions that provide WLH with HPV self-sampling kits.

## Data Availability

The data that support the findings of this study are not openly available due to their containing information that could compromise the privacy of research participants. Deidentified data may be accessible from the corresponding author, D.L., upon reasonable request.

## Abbreviations

U.S.: United States
ICC: Invasive cervical cancer
COVID-19: Coronavirus disease of 2019
HPV: Human papillomavirus
WLH: Woman living with HIV
Pap: Papanicolaou
HIVMA: HIV Medicine Association
IDSA: Infectious Diseases Society of America
WBMA: Washington-Baltimore Metropolitan Area
DC: District of Columbia
HIV: Human immunodeficiency virus
FGD: Focus group discussion(s)
REDCap: Research Electronic Data Capture
SMS: Short message service
